# Reclassifying variations of unknown significance in diseases affecting Saudi Arabia’s population reveal new associations

**DOI:** 10.3389/fgene.2023.1250317

**Published:** 2023-11-01

**Authors:** Mariam M. Al Eissa, Raniah S. Alotibi, Bader Alhaddad, Taghrid Aloraini, Manar S. Samman, Abdulrahman AlAsiri, Mohamed Abouelhoda, Amerh S. AlQahtani

**Affiliations:** ^1^ Public Health Authority, Public Health Lab, Molecular Genetics Laboratory, Riyadh, Saudi Arabia; ^2^ Medical School, AlFaisal University, Riyadh, Saudi Arabia; ^3^ Department of Clinical Laboratory Sciences, College of Applied Medical Sciences, King Saud bin Abdulaziz University for Health Sciences (KSAU-HS), Riyadh, Saudi Arabia; ^4^ King Abdullah International Medical Research Center (KAIMRC), Riyadh, Saudi Arabia; ^5^ Laboratory Medicine Department, King Fahd University Hospital, Imam Abdulrahman Bin Faisal University, Dammam, Saudi Arabia; ^6^ Molecular Genetics Department, King Saud Medical City, Riyadh, Saudi Arabia; ^7^ Division of Translational Pathology, Department of Laboratory Medicine, King Abdulaziz Medical City, Riyadh, Saudi Arabia; ^8^ Department of Genetics, King Abdullah Specialized Children Hospital, King Abdulaziz Medical City, MNGHA, Riyadh, Saudi Arabia; ^9^ Department of Pathology and Clinical Laboratory Medicine Administration, King Fahad Medical City (KFMC), Riyadh, Saudi Arabia; ^10^ Medical Genomics Research Department, King Abdullah International Medical Research Center, King Saud Bin Abdulaziz University for Health Sciences, Ministry of National Guard Health Affairs, Riyadh, Saudi Arabia; ^11^ Department of Cardiology, Division of Heart and Lungs, University Medical Center Utrecht, University of Utrecht, Utrecht, Netherlands; ^12^ Chairman Computational Science Department at King Faisal Specialised Hospital and Research Center, KFSHRC, Riyadh, Saudi Arabia; ^13^ Medical Genetics Department, King Saud Medical City, Riyadh, Saudi Arabia

**Keywords:** single nucleotide variation, variants of unknown significance, exome sequencing, next-generation sequencing, novel variation, disease-related

## Abstract

**Introduction:** Physicians face diagnostic dilemmas upon reports indicating disease variants of unknown significance (VUS). The most puzzling cases are patients with rare diseases, where finding another matched genotype and phenotype to associate their results is challenging. This study aims to prove the value of updating patient files with new classifications, potentially leading to better assessment and prevention.

**Methodology:** We recruited retrospective phenotypic and genotypic data from King Saud Medical City, Riyadh, Kingdom of Saudi Arabia. Between September 2020 and December 2021, 1,080 patients’ genetic profiles were tested in a College of American Pathologists accredited laboratory. We excluded all confirmed pathogenic variants, likely pathogenic variants and copy number variations. Finally, we further reclassified 194 VUS using different local and global databases, employing *in silico* prediction to justify the phenotype–genotype association.

**Results:** Of the 194 VUS, 90 remained VUS, and the other 104 were reclassified as follows: 16 pathogenic, 49 likely pathogenic, nine benign, and 30 likely benign. Moreover, most of these variants had never been observed in other local or international databases.

**Conclusion:** Reclassifying the VUS adds value to understanding the causality of the phenotype if it has been reported in another family or population. The healthcare system should establish guidelines for re-evaluating VUS, and upgrading VUS should reflect on individual/family risks and management strategies.

## Introduction

Rare diseases (RDs) are life-threatening chronic disorders found in small groups of people (MOH, 2023). According to the online database Orphanet, there are more than 7,362–8,120 gene-related to RDs, with a prevalence of one in 2,000 (OMIM, 2023; Orphanet, 2023). A high rate of consanguineous marriages in Saudi Arabia increases the prevalence of autosomal recessive disorders (Alfares et al., 2017; Monies et al., 2017). This consanguinity results in a high level of pathogenic alleles being carried with increasing frequency in the Saudi population (Eissa et al., 2021; Aleissa et al., 2022). The government of Saudi Arabia, represented by the Ministry of Health, has introduced several screening programs to reduce the burden of specific diseases carried by the Saudi population (Saudi Ministry of Health, 2020). However, several studies have reported multiple diseases that share similar phenotypes. For example, a multicenter study of 3,310 clinical exome sequencing (ES) cases performed on 2,219 families characterized several phenotypes. These phenotypes were mainly neurodevelopmental disorders, congenital malformations, dysmorphic features, and neuromuscular dystrophies (Monies et al., 2019). The same phenotypes were also observed in several other studies (Bertoli-Avella et al., 2021a; Bertoli-Avella et al., 2021b; Alotibi et al., 2023) and can still be identified and diagnosed through genetic testing. Other ultra-rare variations are usually challenging to diagnose due to phenotypic variability (Balci et al., 2017; Posey et al., 2017). In ultra-rare cases, it might be associated with an overlap of two phenotypes due to having more genetic associations than a single-gene disorder. In these cases, the diagnosis is a dilemma; practitioners must consider multiple diagnoses as part of an appropriate solution (Armour et al., 2016). However, more than 50% of these cases remain with no diagnosis or treatment, leaving the patients and their families suffering from a prolonged and expensive diagnostic odyssey (Lewis et al., 2010; de Ru et al., 2012). In addition to the frustration caused to patients and their caregivers, this lengthy process also burdens healthcare providers. In the United States, it is estimated that a diagnostic odyssey costs more than US $10,000 for each case (Marshall et al., 2019).

Genomic medicine worldwide has encountered exponential advances in sequencing technology and interpretation tools, accelerating the diagnostic yield for several diseases (Seaby, Rehm, and O’Donnell-Luria, 2021). However, the results remain variants of unknown significance (VUS), and several patients still wait years to be diagnosed genetically (Deignan et al., 2019). Unsolved cases account for two-thirds of all RDs (Tan et al., 2020). While the American College of Medical Genetics and Genomics emphasizes revisiting undiagnosed cases (Deignan et al., 2019), RD patients remain undiagnosed due to limited knowledge of certain variations that might be associated with their phenotypes (Truty et al., 2019). Downstream analysis plays a significant role in variant annotation, data filtering, and technical aspects affecting sequence quality (Tan et al., 2020). This study aims to reclassify VUS and match them with reported phenotypes.

## Methodology

### Ethical approval

Ethical approval was given by the local institutional review board at the King Saud Medical City (KSMC) research center, Ministry of Health, Saudi Arabia (protocol approval number H1R1-01-Aug21-01), in September 2021 for 1 year.

### Sample size and study design

A retrospective study was conducted at KSMC in Riyadh, Kingdom of Saudi Arabia. The inclusion criteria included patients who visited or were referred to KSMC between September 2020 and December 2021 with suspected genetic diseases. The enrolled patients consented to the genetic testing protocol for diagnostic purposes. Overall, 1,080 individuals were tested at a commercial laboratory accredited by the College of American Pathologists (ALqahtani et al., 2023). The analysis used next-generation sequencing (NGS) to perform genome sequencing (GS), exome sequencing (ES), gene panels, mitochondrial GS (Illumina platform), and array-based technology (microarray).

### Data collection

In this study, the variants and their initial classifications were obtained via a thorough review of clinical reports of patients. The recruited data included basic patient demographics, phenotypes, clinical investigations, patient information, family history, and consanguinity collected by a medical geneticist. The patient report included the variation that was most probably associated with the phenotype. Overall, 738 probands, representing one member of each family, were recruited. Patients with confirmed diagnoses via genetic testing and those previously reported by Alqahtani et al. (2023) were excluded to avoid bias. One hundred and ninety-four variants remained; for, 158 probands with unresolved cases with genetic reports identifying VUS were included. We excluded copy number variants (CNVs) from this study.

### Databases

**FIGURE 1:**
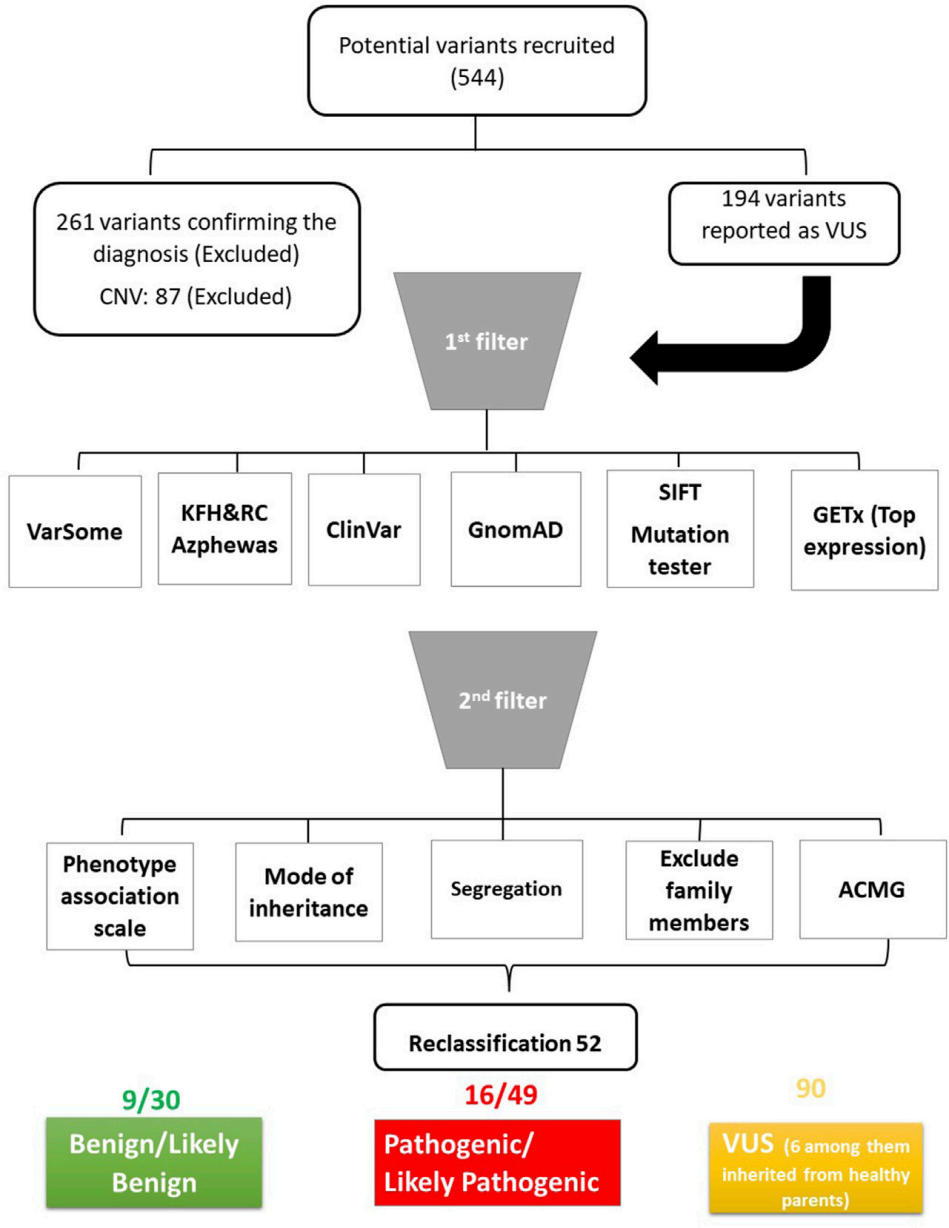
Schematic diagram of the pipeline used to analyse the data. ACMG: American College of Medical Genetics and Genomics*.*

We investigated whether variants classified as VUS had been reported previously to associate them with the same phenotype. For this, we followed the pipeline described in Figure 1, starting with the research platforms VarSome 11.7 and ClinVar (Landrum et al., 2016; Kopanos et al., 2019). Furthermore, we evaluated pathogenicity using several *in silico* databases, including Sorting Intolerant from Tolerant (SIFT) (Ng and Henikoff, 2003) and Mutation Taster (Schwarz et al., 2010). We also investigated allele frequency in gnomAD database (Karczewski and Francioli, 2017). We then checked the expression level using the GETx database to correlate the disease with its top-expressing tissue (Lonsdale et al., 2013). The mode of inheritance was related to the phenotype if it justified the known phenotype inheritance mode. We also investigated segregation to exclude if the variants were segregated in unaffected family members. To examine allele frequency in the Saudi population, we investigated the local in-house database in King Faisal Hospital and Research Centre (KFH&RC), which contains more than 13,000 ES of Saudi individuals. Moreover, we investigated gene-phenotype associations in the repository (Azphewas, 2023) to look for rare variant contributions to human diseases (Wang et al., 2021). Finally, we assessed the phenotype-to-genotype relationship as follows: related to phenotype, could explain the phenotype, could explain part of the phenotype, unrelated to phenotype, and unsure.

## Results

**FIGURE 2:**
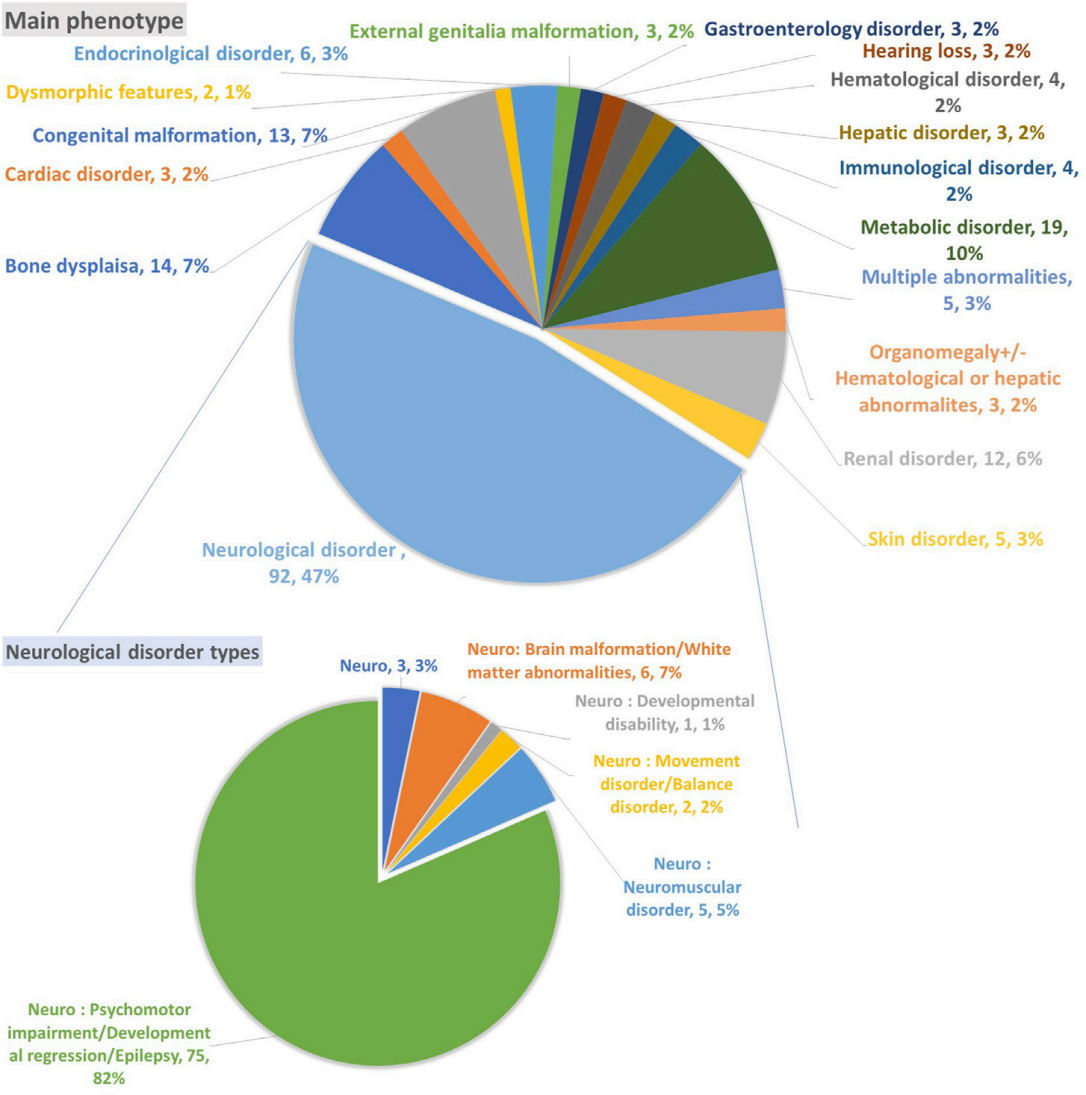
The primary phenotype associated with unsolved cases and variants of unknown significance.

The included participants in this study underwent 186 ES as Solo, two ES as Duo, one ES as Trio, one GS as Trio, eight panels, and three mitochondrial GS (Table 1). Most samples were associated with neurological disorders, accounting for 47% (92 variants); 82% (75 variants) of the neurological disorders were associated with psychomotor impairment, developmental regression, and epilepsy. The second phenotype observed was metabolic disorder (10%; 19 variants), followed by congenital malformation (7%; 13 variants) and bone dysplasia (7%; 14 variants), with a minor representation of other phenotypes, as indicated in Figure 2.

**TABLE 1:** The type of molecular tests according to the reclassified variants.

Classification	ES (Solo)	ES (Duo)	ES (Trio)	GS (Trio)	Panels	Mitochondrial genome (sequencing)
Pathogenic/Likely pathogenic	65*	_	_	1*	Nephrology panel (1)	_
					Metabolic panel (1)	
Uncertain significance	84*	2*	1*	1*	Anaemia panel (2)	3*
					Nephrology panel (2)	
					Immunology panel (1)	
Likely benign/Benign	39*	_	_	_	Autism panel (1)	_

*Subjects can have more than one test.

**FIGURE 3:**
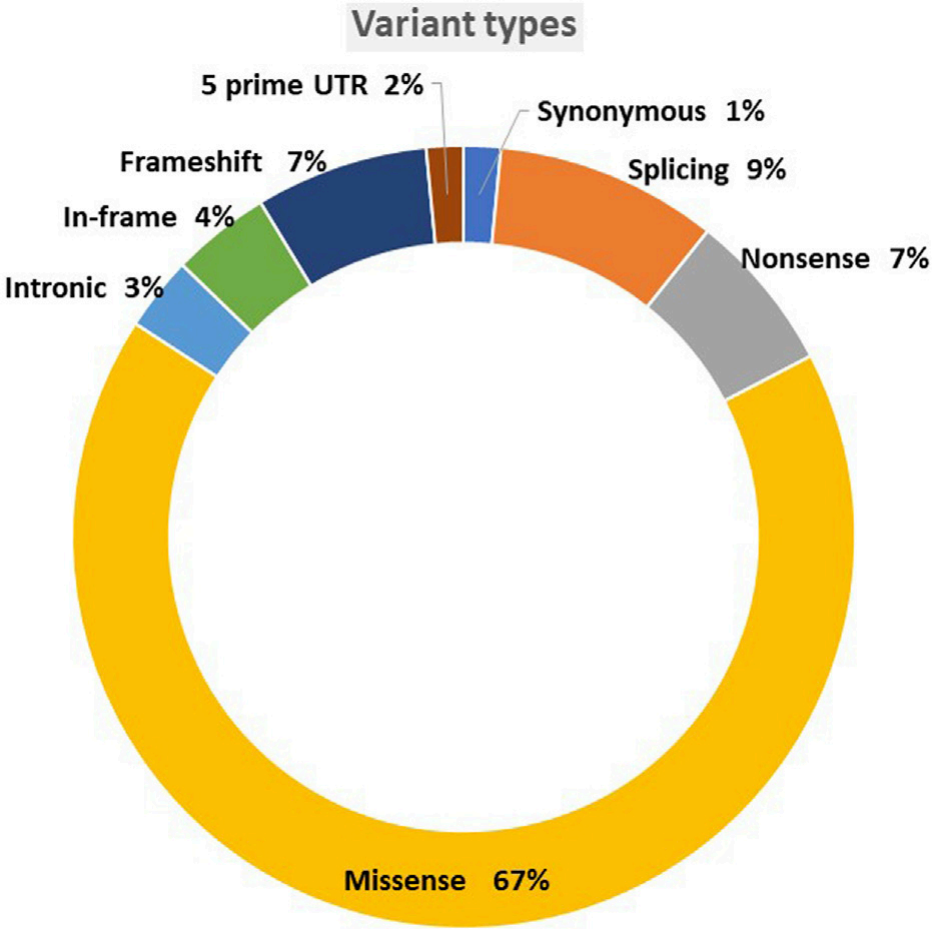
Variant types.

**FIGURE 4:**
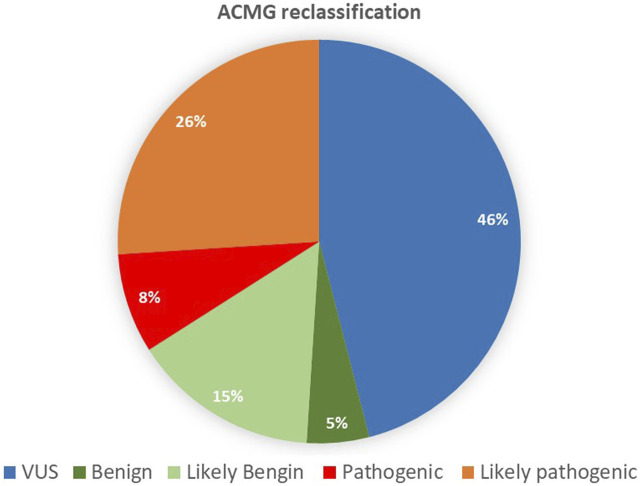
Reclassifications, following the American College of Medical Genetics and Genomics guidelines.

The variants were mainly missense, accounting for 67% of the total single nucleotide variant, followed by 9% splicing and 7% frameshift (Figure 3). Although 90 variants remained VUS out of the original unclassified variants, we reclassified more than half of the original 194 variants. In total, 104 variants were classified with the following reclassifications: nine as benign (B), 30 as likely benign (LB), 16 as pathogenic (P), and 49 as likely pathogenic (LP), as shown in Figure 4.

**FIGURE 5:**
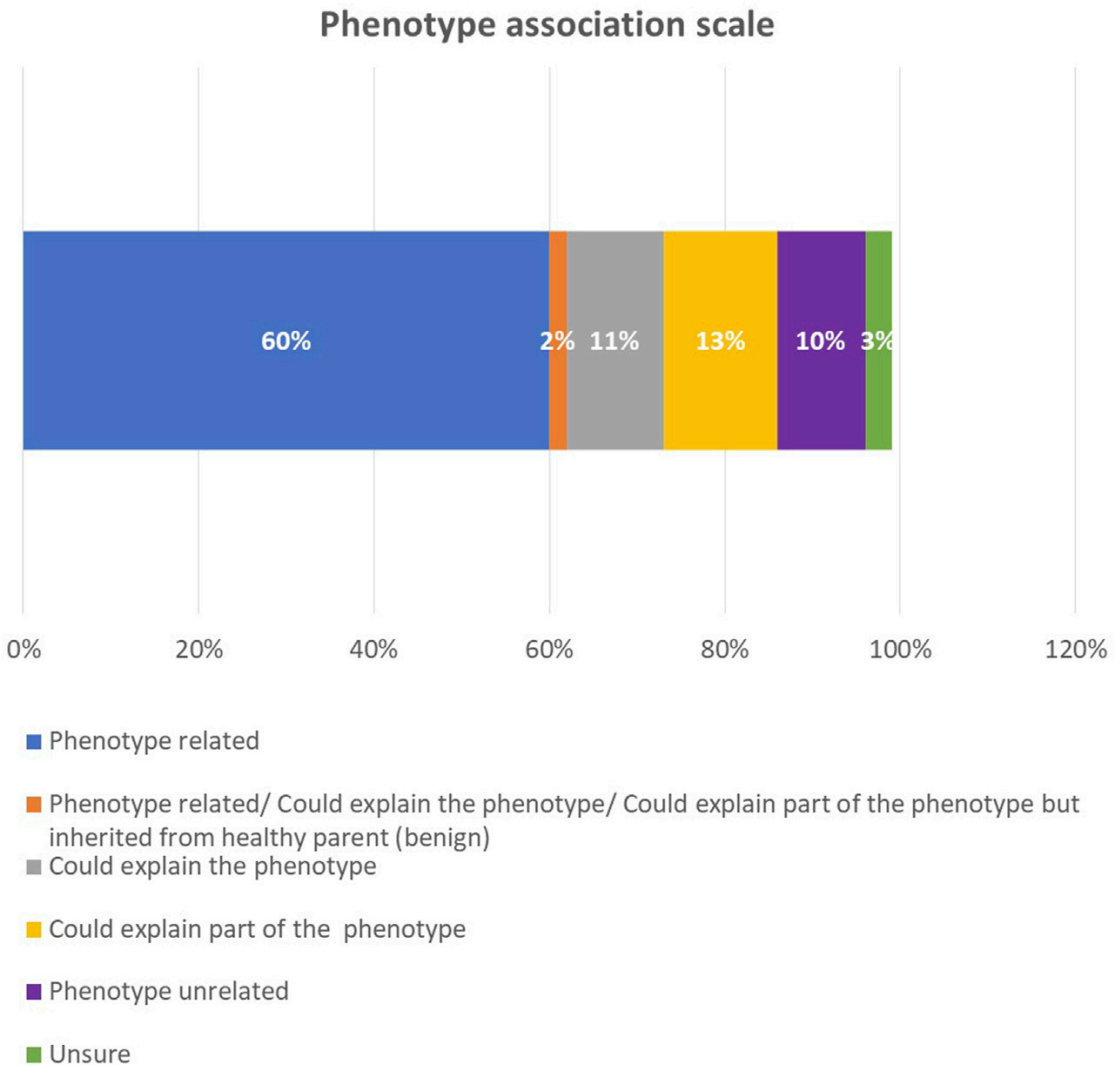
The phenotype association scale among all probands.

After we correlated the phenotype with the genotype in each sample, we found that 60% of the phenotypes were related to a genotype (Figure 5). In 13% of the cases, the genotype explained part of the phenotype; in 11% of the cases, it could explain the phenotype; in 10% of the cases, it was not associated with the phenotype; and in 3%, the correlation remained unsure. Among the positive reclassified cases, the genotype was related to the phenotype in 48 cases (77.4%), could explain the phenotype in six cases (9.6%), and was unrelated to the phenotype in four cases (6.4%). The modes of inheritance among the positive reclassified cases were as follows: autosomal recessive (AR) and autosomal dominant (AD) were approximately equal, with 28 (45%) and 27 (43.5%) cases, respectively, X-linked comprised four cases (6.4%), and AR *versus* AD comprised two cases (3%).

A review of the KFH&RC database illustrated that 11 P, 40 LP, 63 VUS, 17 LB, and 2 B variants had never been previously reported among the Saudi population. In comparison, 5 P, 8 LP, 29 VUS, 13 LB, and 7B variants had been previously noticed in this database. Exploring Azphewas indicated that 12 variants were previously observed; however, there was no strong correlation with a specific phenotype. Details of the 194 variants can be found in Supplementary Tables S1–S4.

## Discussion

According to their relevance, genetic variants can be classified into one of the following categories: P, LP, VUS, LB, and B (Richards et al., 2015). VUS classification has an unknown impact on health, leaving patients and clinicians uncertain about the variant’s pathogenicity (Iancu et al., 2021). Among the 194 reclassified VUS, about 25% were upgraded to LP, and 8% were P. Around 15% of the VUS were downgraded to LB, 5% were B, and the majority remained VUS (49%). Among the VUS variants, six were AD inherited from a healthy parent, which excluded disease associations (Supplementary Table S4). Among the six variants, one was observed to be heterozygous in the KFH&RC database. Exploring the database further revealed that more than 60 VUS variants had never been observed in the Saudi population, and 29 had previously been observed in the dataset.

The reclassifications from VUS to LP and P variants indicated that 51 newly classified variants had never been observed in KFH&RC. Among these 51 variants, 31 had not been observed in ClinVar; thus, they might be novel variants. In all, 13 variants had been previously observed in the KFH&RC database, showing common to rare allele frequencies. The lack of a disease database limits novel variant discovery in Saudi Arabia because the international database lacks Saudi descent (Monies et al., 2019).

Downgrading a VUS to B or LB might concern the requested healthcare provider and/or proband. Hence, further explanation should be provided regarding the variant classification process and the evidence upon which it is based. It may be advantageous to exclude this variant and seek another variation that might not appear in conventional NGS. For instance, identifying new causative variations may require techniques that explore CNVs, including chromosomal microarrays or long-read sequencing (Pauper et al., 2021).

Healthcare providers and patients rely on the accuracy of their variants’ results to make life-changing medical decisions; however, the uncertainty of VUS might create confusion for healthcare providers and patients. Due to a limited understanding of VUS, non-genetics healthcare providers might erroneously interpret and incorporate VUS into management decisions (Kurian et al., 2017). It has been demonstrated that some surgeons have performed bilateral breast mastectomies on patients with unilateral breast cancer with VUS in *BRCA1* and *BRCA2* without consulting a medical geneticist or a genetic counselor. Furthermore, several studies have indicated that patients and their families do not always fully comprehend the uncertainty of a VUS, which might not have been appropriately communicated to them. This confusion compounds the psychological impact experienced by patients when VUS results are disclosed (Gould et al., 2022). A multidisciplinary team, including board-certified medical geneticists, laboratory molecular geneticists, and bioinformation and genetics counselors, should carefully evaluate the reassessment of VUS. This team should re-evaluate the patient’s phenotypes, conduct clinical investigations, establish genotype–phenotype correlations, and determine whether the associated disorder’s attributed variants and clinical features are linked to the patient’s phenotype (Corominas et al., 2022; Friedman et al., 2020). The reassessment of VUS should reflect the individual/family risks, genetics counseling, and management strategies (Makhnoon et al., 2023).

Although the American College of Medical Genetics and Genomics—Association for Molecular Pathology (ACMG-AMP) (Richards et al., 2015) has established guidelines to create consistency in how laboratories use available evidence to establish a variant’s pathogenicity, not all laboratories classify genetic variants the same way. Another approach would be to implement an artificial intelligence (AI) tool in variant interpretation software for continuous and dynamic classification and prioritization of variants. These AI tools show that data-driven approaches can solve more VUS cases than guideline-based approaches and *in silico* prediction tools (Lai et al., 2020; Nicora et al., 2022).

It is important to re-evaluate VUS, as future research will improve the understanding of genes and their associated conditions. Laboratories could share data regarding variant classifications in a disease database to improve the understanding of how specific variants impact health. Valuable time could be saved if all testing laboratories were to study variants collaboratively. Furthermore, healthcare institutes should have robust systems and internal guidelines for re-evaluating VUS that reassess frequency and patient follow-up recommendations (Chiang et al., 2021). Evaluating VUS reclassification’s clinical and psychological impacts on the healthcare system and patients/families is also essential (Chiang et al., 2021).

One of the most important recommendations from this study is that all clinical laboratories should implement an internal system to track all sequence variants identified in each gene and clinical evidence when reported. They are also encouraged to contribute to variant databases, such as ClinVar, including clinical evidence used for the variant classification, to aid in the continued understanding of the impact of human variation.

A limitation of this study is that further investigation must be conducted using functional biology to understand the etiology of the disease and its possible target treatments. In summary, most of the reclassified variants were associated with neurological disorders. Revisiting the genetic data and reclassifying the VUS will aid in diagnosis and shorten patients’ diagnostic odyssey. Furthermore, understanding the genetic background of RDs will provide insight into the advancement of gene therapy and precision medicine and accelerate innovations in the pharmaceutical industry.

## Data Availability

The original contributions presented in the study are publicly available. This data can be found here: https://www.ncbi.nlm.nih.gov/clinvar/, SUB13430259: SCV003926473 - SCV003926482.
